# Maternal Exposure to Housing Renovation During Pregnancy and Risk of Offspring with Congenital Malformation: The Japan Environment and Children’s Study

**DOI:** 10.1038/s41598-019-47925-8

**Published:** 2019-08-09

**Authors:** Noriko Motoki, Yuji Inaba, Takumi Shibazaki, Yuka Misawa, Satoshi Ohira, Makoto Kanai, Hiroshi Kurita, Yozo Nakazawa, Teruomi Tsukahara, Tetsuo Nomiyama, The Japan Environment & Children’s Study (JECS) Group, Toshihiro Kawamoto, Hirohisa Saito, Reiko Kishi, Nobuo Yaegashi, Koichi Hashimoto, Chisato Mori, Shuichi Ito, Zentaro Yamagata, Hidekuni Inadera, Michihiro Kamijima, Takeo Nakayama, Hiroyasu Iso, Masayuki Shima, Yasuaki Hirooka, Narufumi Suganuma, Koichi Kusuhara, Takahiko Katoh

**Affiliations:** 1 Center for Perinatal, Pediatric, and Environmental Epidemiology, Shinshu University School of Medicine, 3-1-1 Asahi, Matsumoto, Nagano, 390-8621 Japan; 2 Department of Neurology, Nagano Children’s Hospital, 3100 Toyoshina, Azumino, Nagano, 399-8288 Japan; 3 Department of Pediatrics, Shinshu University School of Medicine, 3-1-1 Asahi, Matsumoto, Nagano, 390-8621 Japan; 4 Department of Rehabilitation, Nagano Children’s Hospital, 3100 Toyoshina, Azumino, Nagano, 399-8288 Japan; 5 Department of Preventive Medicine and Public Health, Shinshu University School of Medicine, 3-1-1 Asahi, Matsumoto, Nagano, 390-8621 Japan; 6 University of Occupational and Environmental Health, 1-1 Iseigaoka, Yahatanishi-ku Kitakyushu, Fukuoka, 807-8555 Japan; 7 National Center for Child Health and Development, 2-10-1 Okura, Setagaya-ku, Tokyo, 157-8535 Japan; 8 Hokkaido University, Kita 8, Nishi 5, Kita-ku, Sapporo, Hokkaido, 060-0808 Japan; 9 Tohoku University, 2-1 Senryo-machi, Aoba-ku, Sendai, Miyagi 980-8575 Japan; 10 Fukushima Medical University,1 Hikariga-oka, Fukushima-shi, Fukushima, 960-1295 Japan; 11 Chiba University, 1-33 Yayoicho, Inage Ward, Chiba-shi, Chiba, 263-8522 Japan; 12 Yokohama City University, 3-9 Fukuura, Kanazawa-ku, Yokohama, Kanagawa 236-0027 Japan; 13 University of Yamanashi, 1110 Shimokato, Chuo, Yamanashi, 409-3898 Japan; 14 University of Toyama, 2630 Sugitani, Toyama-shi, Toyama, 930-0194 Japan; 15 Nagoya City University, 1 Kawasumi, Mizuho-cho, Mizuho-ku, Nagoya, Aichi 467-8601 Japan; 16 Kyoto University, Yoshida-honmachi, Sakyo-ku, Kyoto, 606-8501 Japan; 17 Osaka University, 1-1 Yamadaoka, Suita, Osaka, 565-0871 Japan; 18 Tottori University, 86 Nishi-cho, Yonago, Tottori, 683-8503 Japan; 19 Hyogo College of Medicine, 1-1 Mukogawa, Nishinomiya, Hyogo, 663-8501 Japan; 20 Kochi University, Okochokohasu, Nankoku, Kochi, 783-8505 Japan; 21 Kumamoto University, 1-1-1 Honjo, Chuo-ku, Kumamoto, 860-8556 Japan

**Keywords:** Developmental biology, Risk factors

## Abstract

There have been no large, nationwide, birth cohort studies in Japan examining the effects of house renovation during pregnancy on congenital abnormality. This study examined the impact of (1) prenatal exposure to house renovation and (2) maternal occupational exposure to organic solvents and/or formaldehyde on the incidence of congenital abnormality. The fixed data of 67,503 singleton births from a large national birth cohort study that commenced in 2011 were used to evaluate the presence of congenital abnormalities and potential confounding factors. We employed multiple logistic regression analysis to search for correlations between maternal exposure to house renovation or organic solvents and/or formaldehyde during pregnancy and such congenital abnormalities as congenital heart disease, cleft lip and/or palate, male genital abnormality, limb defect, and gastrointestinal obstruction. After controlling for potential confounding factors, we observed that house renovation was significantly associated with male genital abnormality (OR 1.81, 95% CI 1.03-3.17, *P* = 0.04) when stratified by congenital abnormality, with no other remarkable relations to house renovation or occupational use of organic solvents and/or formaldehyde during pregnancy. There were also significant correlations for maternal BMI before pregnancy, history of ovulation induction through medication, maternal diabetes mellitus/gestational diabetes mellitus, and hypertensive disorder of pregnancy with an increased risk of congenital abnormality. In conclusion, this large nationwide survey provides important information on a possible association of house renovation during pregnancy with congenital male genital abnormality which needs confirmation in future studies.

## Introduction

Several occupational and environmental agents have been implicated in the etiology of congenital malformations^1^. By fetal exposure through the mother, a teratogenic effect may arise during the organogenesis phase, which is most vulnerable in the first 3–8 weeks of pregnancy in humans. It is also possible that different types of structural malformations share common biological mechanisms and that a given teratogenic factor may lead to various malformations depending on the time window and level of exposure^2,3^.

Housing conditions in Japan have steadily improved like in most developed countries. House renovations may become a source of indoor environmental pollution, with exposure to organic solvents, volatile organic compounds (VOCs), and formaldehyde leading to symptoms of sick house syndrome, which include asthma, eczema, and allergies^4–8^.

Maternal occupational exposure to paints, dyes, glues, and other indoor environmental pollution during renovation may be a risk factor for congenital heart disease (CHD) in offspring^9,10^. The Danish National Birth Cohort (DNBC) study indicated that non-occupational exposure to paint fumes in the home may also be associated with other congenital anomalies in the general population^11^. To our knowledge, no studies have investigated if exposure to materials associated with general house renovation, including organic solvents and VOCs, during pregnancy increases the risk of congenital anomalies in Japan. Accordingly, we conducted a large birth cohort study with the specific objective of examining the impact of (1) prenatal exposure to house renovation and (2) maternal occupational exposure to organic solvents and/or formaldehyde on the incidence of congenital abnormality.

## Results

A total of 67,503 (68.7%) mothers with singleton live births who completed the Japan Environment and Children’s Study (JECS) questionnaire were available for analysis. Until the second/third trimester, the overall rate of mothers who responded to have house renovation/interior finishing or maternal occupational use of organic solvents and/or formaldehyde was 3.1% (2,058) and 9.4% (6,196), respectively. The prevalence of CHD, cleft lip and/or palate, male genital abnormality, limb defect, and gastrointestinal obstruction was 756 (1.1%), 160 (0.24%), 253 (0.74% among male infants), 176 (0.26%), and 45 (0.07%) cases, respectively.

Table 1 summarizes the participants’ characteristics and exposure to house renovation or organic solvents categorized by congenital malformations (no congenital malformations [controls], CHD, cleft lip and/or palate, male genital abnormality, limb defect, and gastrointestinal obstruction). No statistical significances were seen for exposure to house renovation/interior finishing or occupational exposure to organic solvents and/or formaldehyde.

**Table 1:** Characteristics of participants with or without congenital abnormality.

Variable	No congenital abnormality (controls)	Congenital heart disease	Cleft lip and/or palate	Male genital abnormality	Limb defect	Gastrointestinal obstruction	*P*
Participants, n	66,113	756	160	253	176	45	
Maternal age at delivery, years (mean ± SD)	31.3 ± 4.9	31.5 ± 5.1	30.9 ± 4.8	32.0 ± 5.0	31.4 ± 4.9	31.7 ± 5.4	0.11^a^
Maternal age group, n (%)							
<35 years	48,270 (73.0)	548 (72.5)	122 (76.2)	171 (67.6)	128 (72.7)	30 (66.7)	
35 + years	17,843 (27.0)	208 (27.5)	38 (23.8)	82 (32.4)	48 (27.3)	15 (33.3)	0.34
Prepregnancy BMI, kg/m^2^ (mean ± SD)	21.2 ± 3.3	21.4 ± 3.7	21.5 ± 3.8	21.2 ± 3.4	21.7 ± 3.4	20.8 ± 3.2	0.13^a^
Maternal BMI group, n (%)							
Underweight (BMI <18.5)	10,454 (15.8)	144 (19.0)	30 (18.8)	42 (16.6)	26 (14.8)	11 (24.4)	
Normal weight (BMI 18.5–24.9)	48,741 (73.7)	506 (66.9)	105 (65.6)	179 (70.8)	123 (69.9)	32 (71.1)	
Overweight (BMI 25.0 + )	6,918 (10.5)	106 (14.0)	25 (15.6)	32 (12.6)	27 (15.3)	2 (4.4)	<0.001
Highest level of education, n (%)							
Junior high school	2821 (4.3)	35 (4.6)	7 (4.4)	10 (4.0)	8 (4.5)	3 (6.7)	
High school	20,526 (31.0)	225 (29.8)	59 (36.9)	85 (33.6)	56 (31.8)	12 (26.7)	
Vocational school/Junior college	28,037 (42.4)	325 (43.0)	59 (36.9)	104 (41.1)	77 (43.8)	21 (46.7)	
University/Graduate school	14,729 (22.3)	171 (22.6)	35 (21.9)	54 (21.3)	35 (19.9)	9 (20.0)	0.98
Annual household income, n (%)							
<4,000,000 JPY	26,653 (40.3)	309 (40.9)	59 (36.9)	90 (35.6)	73 (41.5)	19 (42.2)	
4,000,000–7,999,999 JPY	32,354 (48.9)	372 (49.2)	82 (51.3)	136 (53.8)	88 (50.0)	22 (48.9)	
8,000,000 + JPY	7,106 (10.7)	75 (9.9)	19 (11.9)	27 (10.7)	15 (8.5)	4 (8.9)	0.89
Maternal smoking during pregnancy, n (%)	2,838 (4.3)	33 (4.4)	7 (4.4)	15 (5.9)	12 (6.8)	2 (4.4)	0.50
Partner’s smoking during pregnancy, n (%)	30,783 (46.6)	351 (46.4)	75 (46.9)	116 (45.7)	89 (50.6)	20 (44.4)	0.94
Maternal drinking during pregnancy, n (%)	1,854 (2.8)	20 (2.6)	4 (2.5)	8 (3.2)	4 (2.3)	1 (2.2)	0.99
Means of pregnancy for current birth, n (%)							
Spontaneous	61,906 (93.6)	704 (93.1)	146 (91.3)	227 (89.7)	158 (89.8)	42 (93.3)	
Ovulation induction through medication	1,690 (2.6)	20 (2.6)	9 (5.6)	11 (4.3)	11 (6.3)	2 (4.4)	
Artificial insemination or *in-vitro* fertilization	2,517 (3.8)	32 (4.2)	5 (3.1)	15 (5.9)	7 (4.0)	1 (2.2)	0.009
Maternal infection during pregnancy, n (%)	14,438 (21.8)	161 (21.3)	31 (19.4)	62 (24.5)	42 (23.9)	11 (45)	0.80
Maternal use of folic acid supplements, n (%)	1,414 (2.1)	23 (3.0)	4 (2.5)	2 (0.8)	5 (2.8)	1 (2.2)	0.35
House renovation/Interior finishing, n (%)	2,058 (3.1)	25 (3.3)	3 (1.9)	13 (5.1)	5 (2.8)	2 (4.4)	0.46
Occupational exposure to organic solvents and/or formaldehyde, n (%)	6,196 (9.4)	67 (8.9)	10 (6.3)	16 (6.3)	11 (6.3)	5 (11.1)	0.22

^a^Differences in maternal age and BMI were assessed with one-way repeated measures of ANOVA followed by post-hoc (Bonferroni) testing.

In multivariate logistic regression analysis after adjustment for covariates, we observed a significant association between house renovation during pregnancy and male genital abnormality (OR 1.81, 95% CI 1.03–3.17, *P* = 0.04) (Table 2). There were no significant relationships for exposure to house renovation/interior finishing or occupational exposure to organic solvents and/or formaldehyde with other congenital abnormalities.

**Table 2:** Multivariate logistic regression analysis for congenital abnormalities versus controls.

Variable	Congenital heart disease (n = 756)			Cleft lip and/or palate (n = 160)			Male genital abnormality (n = 253)			Limb defect (n = 176)			Gastrointestinal obstruction (n = 45)		
	OR	95% CI	*P*	OR	95% CI	*P*	OR	95% CI	*P*	OR	95% CI	*P*	OR	95% CI	*P*
House renovation/interior finishing	1.08	0.72–1.61	0.72	0.60	0.19–1.88	0.38	1.81	1.03–3.17	0.04	0.92	0.38–2.24	0.85	1.47	0.36–6.06	0.60
Occupational exposure to organic solvents and/or formaldehyde	0.95	0.74–1.22	0.69	0.64	0.34–1.22	0.17	0.66	0.40–1.10	0.11	0.64	0.35–1.17	0.15	1.20	0.47–3.04	0.71

Model was adjusted for maternal age, BMI before pregnancy, level of education, annual household income, maternal and partner’s smoking habit, and maternal drinking habit, means of pregnancy, folic acid supplements, and maternal infection during pregnancy.

## Discussion

We herein describe the first large scale study in Japan by nationwide birth cohort study to determine the effects of maternal exposure to house renovation/interior finishing or organic solvents during pregnancy on such major congenital malformations as CHD, cleft lip and/or palate, male genital abnormalities including hypospadias and cryptorchidism, limb defect, and gastrointestinal obstruction. Our results indicate that house renovation during pregnancy increases the risk of male genital abnormality.

In this Japan-wide self-reported survey of singleton live births, the prevalence rates of CHD, cleft lip and/or palate, male genital abnormality, limb defect, and gastrointestinal obstruction were 1.1%, 0.24%, 0.74% (among male infants), 0.23%, and 0.07%, respectively, and comparable to those of previous studies. However, rates can differ among regions and races^1,12–16^.

Housing decoration materials typically contain oil paints, dyes, laminate board, solid wood, wallpaper, resin glue, and plywood, among which various kinds of environmental pollutants have been reported. For example, organic solvents, heavy metals, and VOCs such as benzene, toluene, xylene, styrene, and aldehyde may be emitted from paints and dyes, and formaldehyde, trichloroethylene, and VOCs can be found in boards and plywood^8,17,18^. These contaminants may be released into the indoor air during or after house renovations. A previous study showed that formaldehyde concentrations were significantly higher in post-renovated than in pre-renovated homes, which reduced indoor air quality. Moreover, low-quality decoration materials may release greater amounts of pollutants into indoor air^19^.

Several case-control studies have revealed that mothers exposed to organic solvents during pregnancy exhibited an increased risk of giving birth to a child with a cleft lip or palate^20,21^. However, other reports did not observe a positive association between maternal exposure to organic solvents and orofacial defects, which was in agreement with our findings^22^. A case control study in China described that maternal exposure to house renovations increased the risk of CHD. This relationship was stronger for women who had moved into a newly decorated house^23^. Meanwhile, maternal exposure to organic dyes, lacquers, pigments, and paints during the first trimester of pregnancy was related to a higher incidence of cardiac malformations in the fetus^9,10^. The risk of CHD in offspring was also increased when the mother was exposed to organic solvents during pregnancy^24^. The limited number of studies focusing on an association between maternal occupational exposure and limb defects and gastrointestinal obstructions is insufficient for the assessment of risk. Moreover, such case-controlled studies are retrospective in design and may contain recall bias. Even prospective cohort investigations, such as the DNBC study, might not have fully evaluated causality due to the scarcity of outcomes.

Despite the rarity of this pregnancy outcome, our findings indicated a possible adverse association of house renovation during pregnancy on the outcome of male genital abnormalities. Earlier studies have also demonstrated a significant link for maternal occupational exposure to solvents^25,26^, but the mechanism of this phenomenon is not fully understood. As male reproductive organ development relies on androgens and a balanced androgen-estrogen ratio, it has been suggested that exposure to high levels of chemicals interfering with the production or action of sex hormones may disturb male reproductive tract formation^27^. Indeed, specific ingredients contained in house building materials have suspected hormone-disrupting properties that potentially interfere with the fetal development of reproductive organs^27,28^. Welsh *et al*. found that androgen blocking at the onset of fetal testosterone production in early gestation induced hypospadias in 64% and cryptorchidism in 30% of rats^29^. These findings may at least partially explain the mechanism of male genital abnormality by house renovation during pregnancy.

Although our main result may have been influenced by the large number of subjects, i.e., mass significance, increased sample sizes are often necessary to evaluate such rare outcomes as congenital abnormalities. The JECS sample size was confirmed to contain sufficient analytical power. For instance, to test a hypothesis concerning a disorder with a prevalence of 0.1%, such as Down syndrome, a relative risk of 2.0, and an alpha error of 0.05 while using a cohort in which the proportion of individuals with a high level of exposure to the chemical substance of interest is 25%, a sample of 67,503 participants was required to provide a statistical power of 80%^30^.

This study has several limitations. First, the data regarding house renovations and occupational exposure to organic solvents and/or formaldehyde after having noticed pregnancy were collected from self-reported questionnaires in the second/third trimester of pregnancy and therefore subjective. An additional limitation was an inability to evaluate the precise timing of exposure during the sensitive organogenesis period. Accordingly, we could not associate the onset of congenital abnormalities with the timing of maternal exposure to house renovations and organic solvents and/or formaldehyde. Because our investigation contained very few cases of frequent occupational exposure to organic solvents and/or formaldehyde among responders, we could not assess a dose-response effect on the development of congenital abnormalities. In addition, although housing decoration materials likely produced various kinds of chemical exposure, we could not ascertain precisely which substances caused the congenital abnormalities. Finally, we used the data on abnormalities diagnosed until 1 month after birth, and so congenital disorders diagnosed afterwards were not included. Especially for cryptorchidism, the true number may have been overestimated as some cases descend by 1 year of age.

Despite the above limitations, this is the first study using a large dataset from a Japanese nationwide birth cohort study to examine the influence of house renovation and occupational exposure to organic solvents and/or formaldehyde during pregnancy that controlled for confounders identified by previous reports. It provides important information on a possible association of house renovation during pregnancy with male genital abnormality that will need confirmation in future studies.

## Materials and Methods

### Study design and participants

The data used in this study were obtained from the JECS, an ongoing cohort study that began in January 2011 to determine the effect of environmental factors on children’s health.

In the JECS, pregnant women were recruited between January 2011 and March 2014. The eligibility criteria for participants were: 1) residing in the study area at the time of recruitment, 2) expected delivery after August 1, 2011, and 3) capable of comprehending the Japanese language and completing the self-administered questionnaire. Details of the JECS project have been described previously^31,32^. The present study used the “jecs-ag-20160424” dataset (released in June 2016 and revised in October 2016) containing information on 98,259 singleton live births, along with the supplementary dataset “jecs-ag-20160424-sp1”. Specifically, we focused on data regarding house renovation and the occupational use of organic solvents or formaldehyde during pregnancy as self-described by mothers who responded during their second or third trimester of pregnancy. Maternal medical information regarding additional pregnancy details and medical history was collected from subject medical record transcriptions and used as other covariates.

The JECS protocol was approved by the Institutional Review Board on Epidemiological Studies of the Ministry of the Environment as well as by the Ethics Committees of all participating institutions: the National Institute for Environmental Studies that leads the JECS, the National Center for Child Health and Development, Hokkaido University, Sapporo Medical University, Asahikawa Medical College, Japanese Red Cross Hokkaido College of Nursing, Tohoku University, Fukushima Medical University, Chiba University, Yokohama City University, University of Yamanashi, Shinshu University, University of Toyama, Nagoya City University, Kyoto University, Doshisha University, Osaka University, Osaka Medical Center and Research Institute for Maternal and Child Health, Hyogo College of Medicine, Tottori University, Kochi University, University of Occupational and Environmental Health, Kyushu University, Kumamoto University, University of Miyazaki, and University of Ryukyu. The JECS was conducted in accordance with the Helsinki Declaration and other nationally valid regulations and guidelines. Written informed content was obtained from each participant.

### Data collection

Information on socioeconomic status, smoking habits of mothers and their partner, maternal alcohol consumption, house renovation, and maternal occupational use of organic solvents and/or formaldehyde during pregnancy was collected during the second/third trimester of pregnancy by means of self-reported questionnaires. Maternal anthropometric data before pregnancy, complications and medication during pregnancy, and a history of previous pregnancy were collected from subject medical record transcriptions. Pre-pregnancy BMI was used to evaluate maternal weight status and was calculated according to World Health Organization Standards as body weight (kg)/height (m)^2^.

### Outcomes, exposure, and covariates

**Figure 1:**
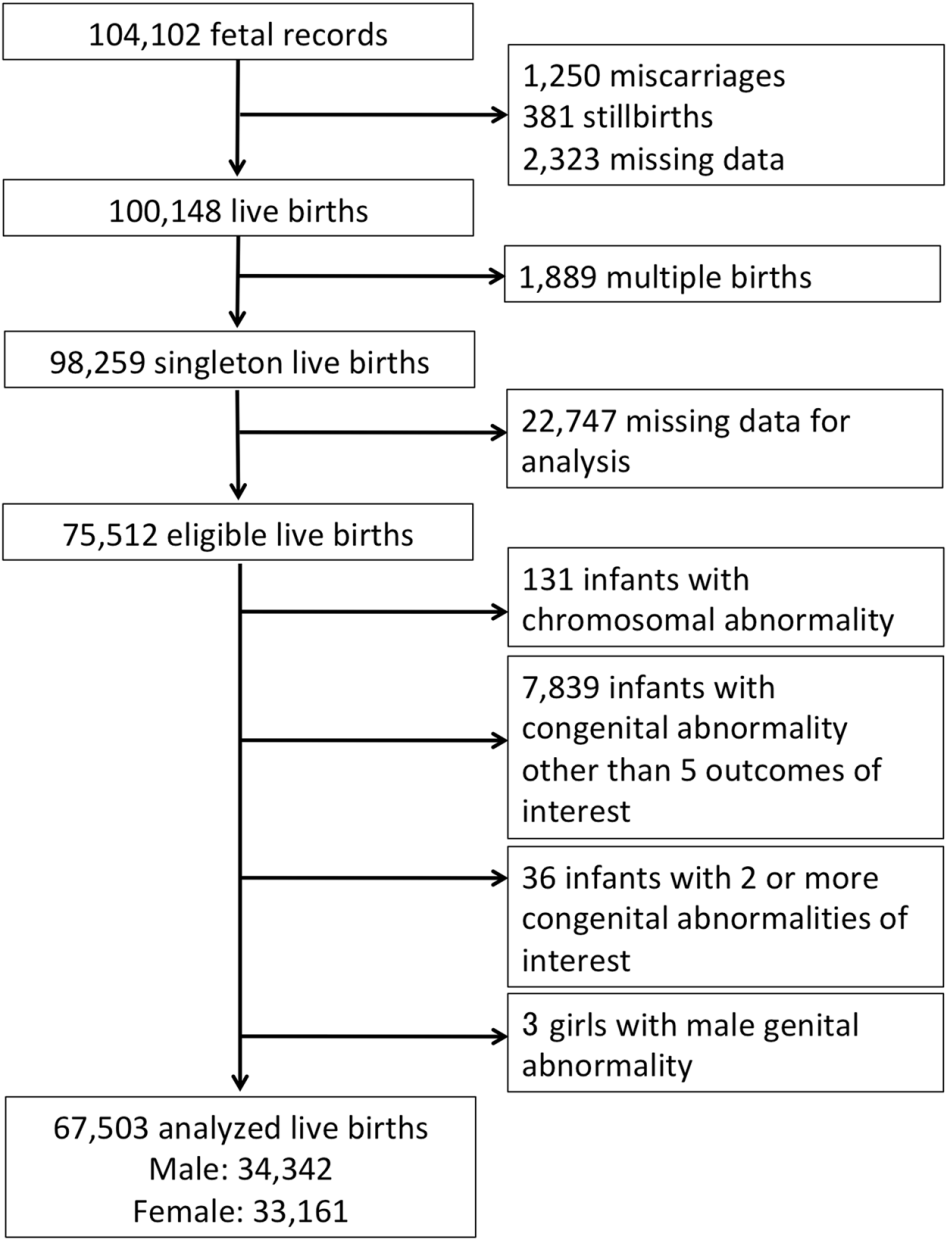
Case selection flowchart.

The main outcomes of interest were congenital malformations diagnosed by the subjects’ obstetricians up to 1 month after birth. We selected diseases that were identifiable by the first month of life and had a relatively high incidence rate among congenital abnormalities: CHD, cleft lip and/or cleft palate, male genital abnormalities such as hypospadia and cryptorchidism, limb defects such as polydactyly, syndactyly, and cleft finger/foot, and gastrointestinal obstructions such as esophageal, duodenal, and small intestinal atresia and imperforate anus. We excluded participants with such chromosomal abnormalities as trisomy 21, trisomy 18, trisomy 13, and Turner syndrome. Subjects with congenital malformations other than the above 5 outcomes of interest or who were afflicted by 2 or more congenital malformations were excluded as well (Fig. 1) to assess the impact of exposure on each disease group. Infants with no congenital abnormality were employed as controls.

The factors of house renovation/interior finishing and maternal occupational use of organic solvents and/or formaldehyde after becoming pregnant were assessed in the second/third trimester of pregnancy. Maternal exposure to house renovation/interior finishing during pregnancy was examined by the questionnaire item of “Did your household undertake house renovation/interior finishing after becoming pregnant?” Examples of organic solvents were paint thinner, solvents for examination/analysis/extraction, dry-cleaning detergents, stain-removing agents, paints, nail polish remover, and others. The frequency of maternal occupational exposure to organic solvents and/or formaldehyde during pregnancy was assessed by the questionnaire item “Please choose the frequency that best describes the use or handling of organic solvents and/or formaldehyde during work for more than half a day after becoming pregnant” and was grouped as “never”, “once to three times a month”, “once to six times a week”, or “every day”. We subsequently divided mothers into unexposed and exposed groups.

Demographic covariates included maternal age, smoking habit of mothers and their partner, maternal alcohol consumption, pre-pregnancy BMI, and socioeconomic status. Socioeconomic status was evaluated by the highest level of education completed by the mother (junior high school, high school, vocational school/junior college, or university/graduate school) and annual household income (<4,000,000, 4,000,000–7,999,999, or 8,000,000 + JPY). Obstetric and medical variables, such as means of pregnancy, maternal infection and medications during pregnancy, were also evaluated.

### Statistical analysis

All statistical analyses were performed using SPSS statistical software version 24 (SPSS Inc., Chicago, Illinois). Differences in maternal age and pre-pregnancy BMI among the types of congenital malformations were assessed by one-way repeated measures of analyses of variance (ANOVA) followed by post-hoc (Bonferroni) test. We categorized all continuous and ordinal variables, such as maternal age (<20, 20–34, or 35 + years), pre-pregnancy BMI (<18.5, 18.5–24.9, or 25 + kg/m^2^), and annual household income. Analysis of variance and chi-square tests were conducted to compare covariates between groups stratified by category as well as by the presence of house renovation/interior finishing or maternal occupational use of organic solvents during pregnancy. We employed logistic regression models to calculate adjusted odds ratios (ORs) and their 95% confidence intervals (CIs). To employ logistic regression models on male genital abnormality, we analyzed subjects limited to male singleton infants. The covariates in our models were selected *a priori* based on previously published literature and biologic plausibility. In model 1, we first estimated the effects of house renovation/interior finishing and maternal occupational use of organic solvents after adjusting for maternal background (age [<35 or 35 + years], pre-pregnancy BMI, smoking and drinking habits. In model 2, we adjusted for the variables in model 1 along with maternal obstetric information (means of current pregnancy), pregnancy complications (maternal infection), and medications (folic acid supplements).
